# Oxidosqualene Cyclase Knock-Down in Latex of *Taraxacum koksaghyz* Reduces Triterpenes in Roots and Separated Natural Rubber

**DOI:** 10.3390/molecules24152703

**Published:** 2019-07-25

**Authors:** Nicole van Deenen, Kristina Unland, Dirk Prüfer, Christian Schulze Gronover

**Affiliations:** 1 Institute of Plant Biology and Biotechnology, University of Muenster, Schlossplatz 8, 48143 Muenster, Germany; 2 Fraunhofer Institute for Molecular Biology and Applied Ecology IME, Schlossplatz 8, 48143 Muenster, Germany

**Keywords:** pentacyclic triterpenes, polyisoprenes, *Taraxacum koksaghyz*, RNA interference, oxidosqualene cyclases

## Abstract

In addition to natural rubber (NR), several triterpenes are synthesized in laticifers of the Russian dandelion (*Taraxacum koksaghyz*). Detailed analysis of NR and resin contents revealed different concentrations of various pentacyclic triterpenes such as α-, β-amyrin and taraxasterol, which strongly affect the mechanical properties of the resulting rubber material. Therefore, the reduction of triterpene content would certainly improve the industrial applications of dandelion NR. We developed *T. koksaghyz* plants with reduced triterpene contents by tissue-specific downregulation of major laticifer-specific oxidosqualene cyclases (OSCs) by RNA interference, resulting in an almost 67% reduction in the triterpene content of NR. Plants of the T1 generation showed no obvious phenotypic changes and the rubber yield also remained unaffected. Hence, this study will provide a solid basis for subsequent modern breeding programs to develop Russian dandelion plants with low and stable triterpene levels.

## 1. Introduction

In Russian dandelion (*Taraxacum koksaghyz*), high concentrations of secondary metabolites such as natural rubber (NR) are found mainly in the latex of root laticifers [1,2]. NR is an important industrial biopolymer that is used for the production of more than 40,000 consumables, including tires and medical devices [3,4]. To date, the main source for high-quality NR is the rubber tree *Hevea brasiliensis*, but increased raw material demand has led to the development of Russian dandelion as an alternative and sustainable rubber source.

*T. koksaghyz* is a self-incompatible, sexually reproductive plant. Consequently, the resulting offspring is heterogenic [4,5], leading to trait divergence among individual plants. One such example is the highly variable triterpene content of dandelion-derived NK, which negatively affects NR properties [6,7]. For this reason, genetic engineering of the corresponding triterpene biosynthesis could support the development of more uniform accessions with low and stable triterpene contents in their roots.

**Figure 1:**
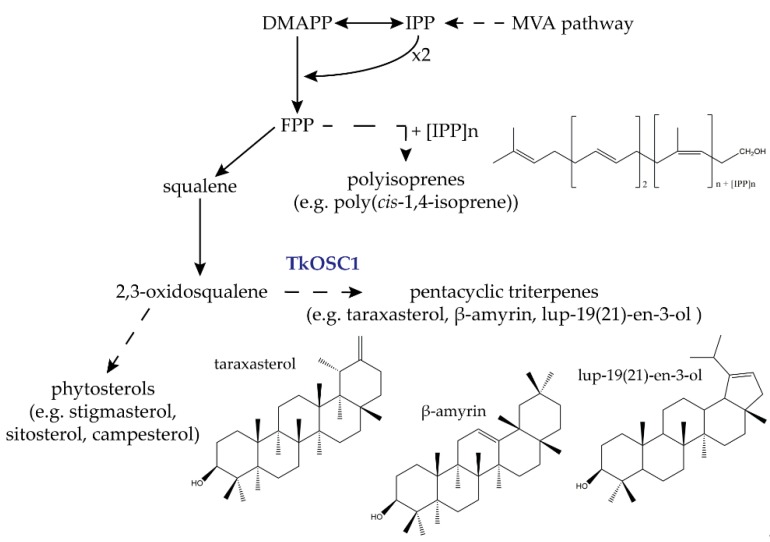
Isoprenoid biosynthesis pathway in *T. koksaghyz* latex. *T. koksaghyz* oxidosqualene cyclase 1 (*TkOSC1*) is a multifunctional OSC that catalyses the formation of several highly abundant pentacyclic triterpenes such as taraxasterol, α-, β-amyrin and lup-19(21)-en-3-ol [9]. Dashed arrows indicate multiple enzyme steps. MVA, mevalonate; DMAPP, dimethylallyl diphosphate; IPP, isopentenyl diphosphate; FPP, farnesyl diphosphate.

The central isopentenyl diphosphate (IPP) building block for triterpenes as well as for poly(*cis*-1,4-isoprenes) such as dolichols or the long-chain NR-forming poly(*cis*-1,4-isoprene) is synthesized by the mevalonate (MVA) pathway in the latex of laticifers (Figure 1) [8,9].

Oxidosqualene cyclases (OSCs; E.C. 5.4.99) catalyse the cyclisation of 2,3-oxidosqualene [10,11], which leads to the production of triterpenes, compounds of 30 carbon atoms, that comprise nearly 100 different scaffolds in plants [12]. Triterpenes can be further modified by tailoring enzymes via acetylation, oxidation or by adding sugar chains, resulting in a huge diversity of more than 20,000 known triterpenoids [11,12]. Due to the cyclisation reaction type, two groups of triterpenes can be formed: The first group is characterised by a chair–boat–chair conformation resulting in the production of cycloartenol, which is a phytosterol intermediate [10,11]. Phytosterols function, for example, as membrane compounds or signalling molecules, making them important for plant development and growth [13]. In the second group, a chair–chair–chair conformation results in the formation of pentacyclic triterpenes such as lupeol, β-amyrin and taraxasterol among others [11]. A large number of pentacyclic triterpenes show antimicrobial and antifungal characteristics and play a role in plant defence. Due to these characteristics, they are interesting for applications in food, cosmetics and pharmaceutical industries [14,15,16].

Recently, we provided a comprehensive analysis of the triterpene composition in NR extracts from *T. koksaghyz* and identified 13 triterpenes and triterpenoids, also including the so far unknown pentacyclic compound lup-19(21)-en-3-ol [17]. Furthermore, we isolated and functionally characterized seven different *T. koksaghyz* OSCs (*TkOSC1-6* and TkLUP) and could demonstrate that *TkOSC1* is highly expressed in laticifers and represents a multifunctional OSC capable of synthesizing at least four of the latex-predominant pentacyclic triterpenes (taraxasterol, α-, β-amyrin and lup-19(21)-en-3-ol) [17,18] (Figure 1).

In this study, we developed transgenic *T. koksaghyz* plants with reduced levels of pentacyclic triterpenes by laticifer-specific downregulation of *TkOSC1* expression via RNA interference. Lower amounts of triterpenes were also evident in extracted rubber, which certainly will contribute to a significant improvement of the resulting material properties.

## 2. Results

### 2.1. Generation of T. koksaghyz OSC RNAi Plants

**Figure 2:**
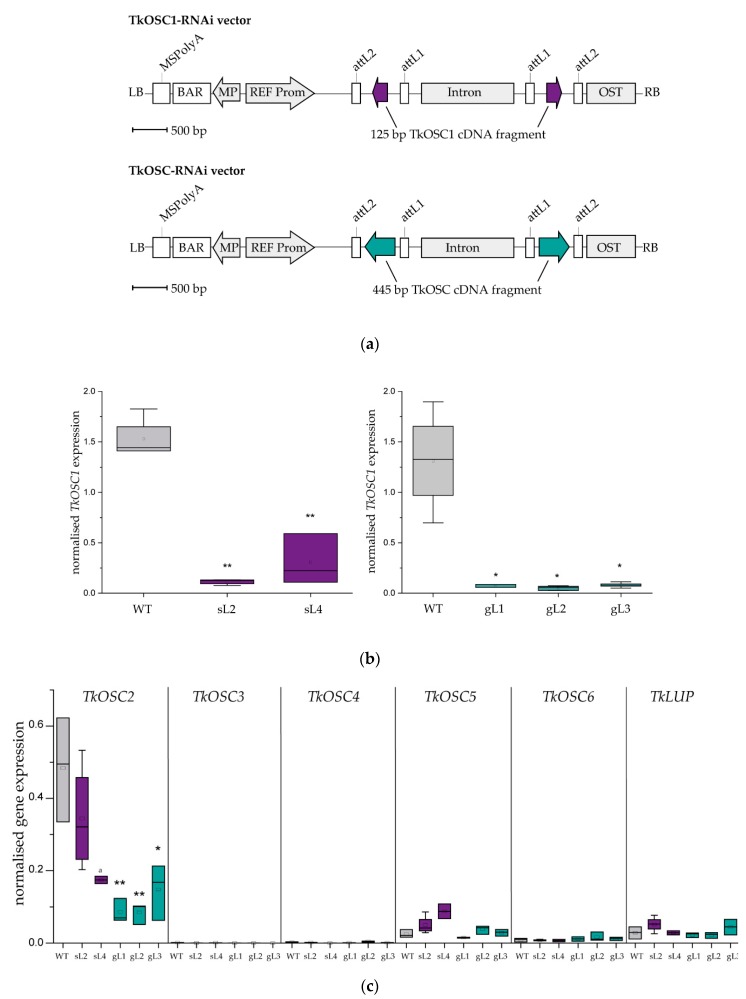
RNA silencing of *TkOSCs* in *T. koksaghyz*. (**a**) Schematic representation of *TkOSC1*- and *TkOSC*-RNAi vectors. A 125 bp cDNA fragment deriving from a non-conserved region was supposed to knock down *TkOSC1* expression only, whereas a 445 bp-cDNA fragment derived from a more conserved region was expected to knock down several *TkOSCs*. REF Prom: laticifer-predominant promoter of the rubber elongation factor (REF); *att*L1/2: *att*L cassette frame; BAR: basta resistance gene; Intron: chalcone synthase intron from *P. hybrida*; LB: left border; MP: mannopine synthase promoter; MSPolyA: mannopine synthase poly(A) signal; OST: octopine synthase poly(A) signal; RB: right border; (**b**) and (**c**) Quantitative real-time PCR of *TkOSCs* in latex from roots of transgenic *TkOSC1*-RNAi lines (sL), *TkOSC*-RNAi lines (gL) and wild-type (WT) plants; the expression levels were normalized to the constitutive genes encoding elongation factor 1a (*TkEF1a*) and ribosomal protein L27 (*TkRP*) from *T. koksaghyz*. Asterisks denote statistical significance compared to control (two-tailed t test, * *p* < 0.05, ** *p* < 0.01). (**b**) normalized *TkOSC1* expression in wild-type and *TkOSC*1/*TkOSC*-RNAi plant lines (*n* = 3–8). (**c**) normalized expression of further *TkOSC* genes in all transgenic lines compared to WT (*n* = 2–4), ^a^ *n* = 2.

In order to generate *T. koksaghyz* plants with reduced triterpene levels in the latex, two strategies were applied. For *TkOSC1* knock-down, a RNAi construct directed against a 125 bp *TkOSC1*-specific region was developed and designated as a *TkOSC1*-RNAi vector (Figure 2A). In contrast, a parallel knockdown of several *TkOSCs* should be achieved by a RNAi vector containing a 445 bp sequence displaying a more conserved region of OSCs (designated as *TkOSC*-RNAi vector, Figure 2a). The expression of the resulting constructs was controlled by the predominant laticifer-specific promoter of the rubber elongation factor gene (REF) [19] (Figure 2a).

Both constructs were transformed into *T. koksaghyz* plants using agrobacterium-mediated plant transformation. After the initial expression analyses, two lines (sL2 and sL4) for *TkOSC1*-RNAi and three lines (gL1-3) for *TkOSC*-RNAi with strongest *TkOSC1* reduction as well as *T. koksaghyz* wild-type (WT) plants at the same developmental stage (12 weeks after transferring from sterile culture into soil) were used for quantitative gene expression studies. As shown in Figure 2b the expression of *TkOSC1* was significantly reduced in the latex in all transgenic lines, with only 15% for line sL2 and 8% for line gL2 of the wild-type expression level. Selected plants (*n* = 2–4) from all transgenic lines were then analysed regarding their expression of other known OSC genes (*TkOSC2-6* and TkLUP [17]) in the latex (Figure 2c). In *TkOSC*-RNAi lines (gL), the expression of another predominant latex OSC gene *(TkOSC2*, [17]) was significantly reduced. *TkOSC2* expression was also slightly reduced in sL2 and sL4 plants, which results obviously from the partial sequence similarity with *TkOSC1*. All other OSC genes showed very low expression in the latex of transgenic and WT plants, varying between 0.001 and 0.05 normalized gene expression. Only *TkOSC5* was slightly upregulated in *TkOSC1*-RNAi plants with a normalized expression of 0.08 in the case of sL4. Overall, we were able to achieve the downregulation of latex-predominant OSC genes with both RNAi constructs, and we achieved a stronger effect in plants carrying the *TkOSC*-RNAi construct.

### 2.2. Laticifer Predominant OSC Knockdown Results in Triterpene Depleted NR

**Figure 3:**
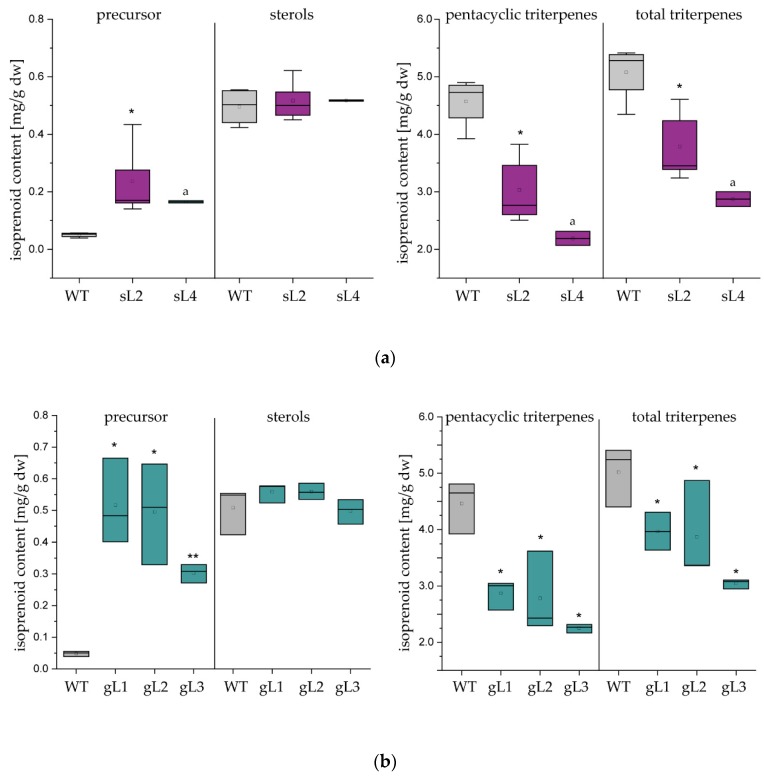
Isoprenoid analysis in freeze-dried root material from 6-month-old (**a**) *TkOSC1*-RNAi and (**b**) *TkOSC*-RNAi plant lines by GC–MS compared to WT plants (*n* = 2–5), ^a^ *n* = 2; asterisks denote statistical significance compared to control (two-tailed t test, * *p* < 0.05, ** *p* < 0.01); precursor: triterpene (squalene and 2,3-oxidosqualene) and sterol precursor (cycloartenol and 24-methylene cycloartanol); sterols: campesterol, stigmasterol and sitosterol; pentacyclic triterpenes: taraxasterol, α- and ß-amyrin, lup-19(21)-en-3-ol, lupeol, taraxerol and four unknown triterpenes; total triterpenes: precursor, sterols and pentacyclic triterpenes; single triterpene levels are shown in Tables S1 and S2.

To analyse whether the OSC knockdown has an impact on triterpene composition in the RNAi lines, two to five plants from all transgenic lines and WT plants were harvested after 6 months of cultivation and triterpene extracts from dry root material were analysed by GC–MS (Figure 3a,b, Tables S1 and S2). The sterol content including campesterol, stigmasterol and sitosterol was unaffected in all RNAi lines, thereby confirming that the RNAi constructs predominantly affected OSC gene expression in laticifers since those phytosterols mainly occur in adjacent root cells, but not in laticifers. In contrast, the most dominant pentacyclic triterpenes of the latex such as β-amyrin and taraxasterol [17] were remarkably reduced in all transgenic RNAi lines. As an additional effect, we detected a significant increase of precursor which was mainly the result of a weak accumulation of the intermediate compounds cycloartenol and 24-methylene-cycloartanol; these sterol precursors were not found in WT roots. However, a triterpene reduction up to 60% in root tissue was detected compared to the WT material.

Next, rubber extracts of root material from three *TkOSC1*-RNAi and *TkOSC*-RNAi plants with the highest reductions in triterpene level compared to WT plants were analysed in order to study the potential impact of the total triterpene reduction on the triterpene content in NR. Triterpenes of the separated NR were extracted by acetone and quantified in relation to NR weight (Table 1). The amount of pentacyclic triterpenes were highly decreased in NR, especially in the material from the *TkOSC*-RNAi lines, leading to a reduction of up to 72%. Interestingly, a considerable amount of precursor as well as sterol compounds were also detectable in NR extracts, indicating that those triterpenes were also attached to the rubber polymer during processing. Nevertheless, a reduction of up to 56% of total triterpenes in NR could be achieved by downregulating OSC genes in laticifers.

### 2.3. Pentacyclic Triterpene Depletion has no Impact on Plant Development and Rubber Yield

**Figure 4:**
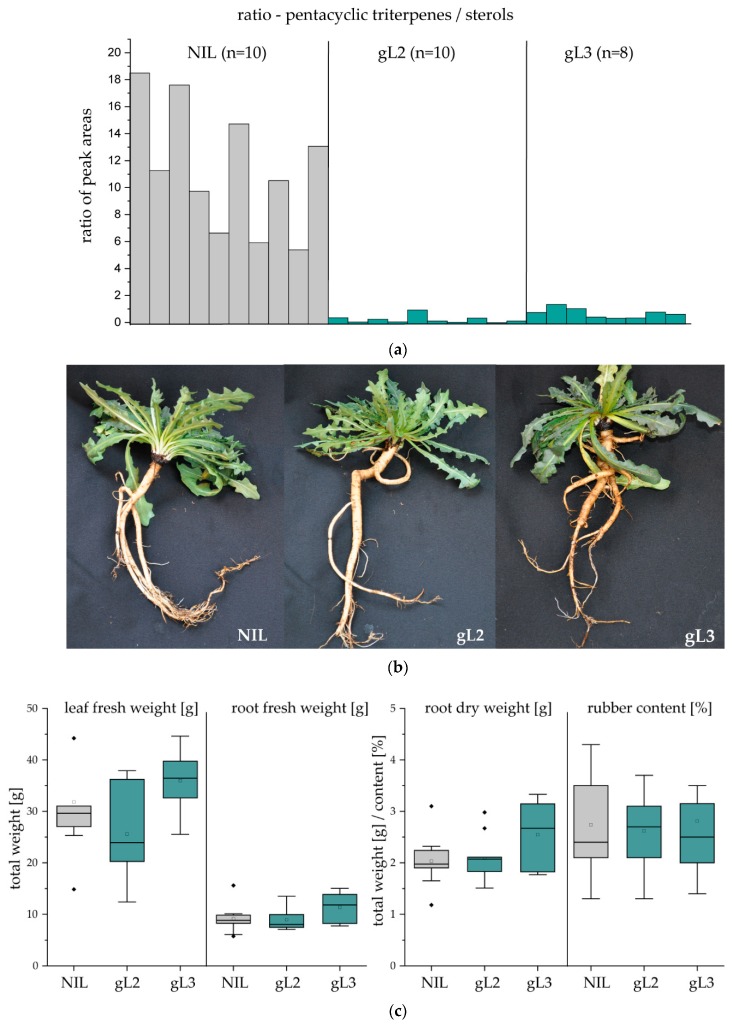
Analysis of *TkOSC*-RNAi plant lines gL2 and gL3 from the T1-generation in comparison to near isogenic lines (NIL). (**a**) Screening of T1 plants by fast triterpene extraction from root material; the ratio of the dominant triterpene end-products (α-, β-amyrin and taraxasterol) and sterols (campesterol, stigmasterol and sitosterol) was calculated in acetone extracts analysed by GC–MS; (**b**) representative pictures of 12-week-old plants after harvesting; (**c**) quantification of leaf and root fresh weight, root dry weight and rubber content (determined by ^1^H-NMR analysis) of 12-week-old plants (NIL and L2, *n* = 10; L3, *n* = 8); no statistical significant differences could be observed.

We next analysed the impact of the RNAi-mediated OSC knockdown on plant development and rubber yield in the following generation. For seed generation, transgenic plants were pollinated with one compatible *T. koksaghyz* WT plant, and offspring plants of two *TkOSC*-RNAi-lines (gL2 and gL3) were subjected to further analysis. Plants that did not contain the transgene (near isogenic lines; NIL) served as controls. To screen the transgenic plants regarding the OSC knockdown effect we established a fast triterpene extraction and detection method. Acetone extracts from about 10 mg dry root material were analysed by GC–MS, and the ratio of the dominant pentacyclic triterpene end-products (α-, β-amyrin and taraxasterol) and the sterols (campesterol, stigmasterol and sitosterol) was calculated (Figure 4a). Using this screening method, 10 gL2-plants and 8 gL3-plants with the most severe knockdown effect were chosen for phenotypical and metabolite analyses and compared to 10 NIL-plants. No developmental changes regarding growth, flowering time, leaf shape or root architecture could be observed between RNAi and control plants (as representative examples, one plant per line and one NIL-plant are shown in Figure 4b). After harvesting the plants at an age of 12 weeks, the leaf fresh weight as well as the fresh and dry weight of roots were analysed, and no significant differences were observed. Furthermore, we found that the triterpene depletion had no impact on the rubber content in the harvested root material (Figure 4c).

In order to confirm the triterpene depletion in NR in the T1-generation, triterpenes were isolated from NR of roots from two gL2-, three gL3- and NIL plants by acetone extraction and quantified in relation to NR weight (Table 2). As a result, the amount of pentacyclic triterpenes was highly decreased, especially in NR from gL2-plants with a reduction of up to 80%. Overall, the total triterpene content in NR could be reduced by about 67% without affecting plant development and NR yield.

## 3. Discussion

In previous studies, we identified and functionally characterised different OSCs involved in triterpene biosynthesis in dandelion [17,18]. In particular, we showed that *TkOSC1* is predominantly expressed in the laticifers, where it catalyses the biosynthesis of several pentacyclic end-products that can be found in NR extracts. In this study, we aimed to generate *T. koksaghyz* plants with reduced levels of triterpenes in the NR by laticifer-specific RNAi either against *TkOSC1* only or several *TkOSCs* in parallel. To achieve this, we first chose a highly specific 125bp-cDNA fragment for *TkOSC1* knock down. A successful downregulation of a particular plant *OSC* gene by RNA interference with a remarkable effect was already shown in the study of Han and co-workers [20,21], who specifically silenced a dammarenediol synthase gene (*DDS*) in *Panax ginseng* resulting in a reduction of ginsenoside production in roots. Furthermore, RNA interference was successfully used in different studies to reduce phytosterol content by silencing *OSC* genes encoding for cycloartenol synthase (CAS), which catalyse the cyclization of 2,3-oxidosqualene into cycloartenol [22,23].

A broader and more unspecific downregulation of all *TkOSC* sequences was achieved in this study with a longer cDNA sequence (445bp) representing a conserved OSC region that contains repeating QW motifs involved in stabilizing carbocationic intermediates [17]. The higher specificity of the *TkOSC1*-RNAi construct in this study could be proven by quantitative expression analyses. While *TkOSC1* expression was remarkably reduced, no significant downregulation of other *TkOSCs* could be detected, and *TkOSC5* was even slightly upregulated in *TkOSC1*-RNAi plant line 4, indicating regulatory feedback mechanisms, which was also observed in *Panax ginseng* plants expressing specific DDS-RNAi constructs [20,21]. *TkOSC2* was slightly reduced in *TkOSC1*-RNAi plants, but no enzyme activity has been detected for *TkOSC2* so far [17], thus it is most likely a pseudogene of *TkOSC1*. In contrast, the expression of the *TkOSC-*RNAi construct resulted not only in a more severe downregulating effect of *TkOSC1*, but also in a significant reduction of *TkOSC2*, the second predominantly expressed *OSC* isoform in latex. Since all other *OSCs* were only detectable at a maximum of 0.05 normalized expression in latex, the overall reduction of *OSC* expression by RNAi was successful.

The remarkable impact of the RNAi effect on triterpene biosynthesis was shown by quantifying the single triterpene levels in whole root tissue as well as in NR extracts. In contrast to the phytosterols (stigmasterol, campesterol and sitosterol), which are mainly present in surrounding root tissue cells and which were desirably unaffected, the amount of triterpenes such as β-amyrin and taraxasterol was highly decreased. The use of a predominantly laticifer-specific promoter [19] led to a significant reduction in the specialized class of pentacyclic triterpenes that are highly abundant in laticifers as well as in NR [17], independent of the cDNA fragment in the RNAi vector. The successful reduction of triterpene end-products in a specialized tissue by RNA interference was already shown in the study of Takagi and coworkers [24], who decreased the saponin content in soybean seeds by expressing RNAi vectors targeting a *Glycine max* β-amyrin synthase gene (*GmBAS1*) under the control of a seed-specific promoter. The high specificity and activity of the REF promoter was also shown in a previous study, where we were able to enhance the isoprenoid biosynthesis in dandelion latex by overexpressing three key enzymes of the mevalonate pathway under the control of this promoter [25]. Using this strategy, it was possible to generate *T. koksaghyz* plants with a highly deregulated triterpene biosynthesis without affecting plant development, as shown in this and previous studies.

In order to establish the Russian dandelion as a valid alternative rubber source, the precise control of the triterpene content in NR is important. In future studies, we will focus on generating *T. koksaghyz* plants with depleted triterpene levels by inducing site-specific knock-outs of the *TkOSC1* gene via genome editing or mutagenesis, and we will characterize in detail the properties of triterpene-depleted NR regarding processability and mechanical properties.

## 4. Materials and Methods

### 4.1. Plant Material and Cultivation Conditions

*T. koksaghyz* wild-type and transgenic plants were grown in standard soil (ED73 Einheitserde, Fröndenberg, Germany). Every 4 weeks, a commercial fertilizer was supplemented according to the manufacturer’s recommendations (Hakaphos Plus, Compo GmbH, Münster, Germany). They were cultivated in controlled growth chambers or in a greenhouse at 18 °C and 20 klux (high-pressure sodium lamp, HPS 600 Watts, Greenbud, enhanced yellow and red spectrum) with a 16-h photoperiod.

### 4.2. Cloning and Transformation Procedures

For cloning of RNAi vectors for stable transformation, cDNA fragments were inserted initially into the Gateway-compatible entry vector pBluescript II KS (+) (Addgene, Cambridge, MA, USA). Therefore, cDNA fragments *TkOSC1*-RNAi (125 bp) and *TkOSC-*RNAi (445 bp) were amplified from latex cDNA by using the oligos *TkOSC1*-RNAi-fwd-NcoI and *TkOSC1-*RNAi-rev-XhoI or *TkOSC-*RNAi-fwd-NcoI and *TkOSC-*RNAi-rev-XhoI, respectively (Table S5). The insertion of the RNAi fragments by using the NcoI/XhoI restriction sites of pBluescript II KS (+) resulted in the entry vectors pBluescript-*TkOSC1*-RNAi and pBluescript-*TkOSC*-RNAi. Subsequently, the entry vectors were used for recombination into the expression vector pLab12.5-pREF [26] resulting in the expression vectors pLab12.5-pREF-*TkOSC1*-RNAi and pLab12.5-pREF-*TkOSC*-RNAi vectors. The integrity of all vector sequences was verified by sequencing. The transformation of *T. koksaghyz* by *A. tumefaciens* strain EHA105 with RNAi constructs was carried out as previously described [27].

### 4.3. Quantitative Expression Analysis

For expression analysis, latex was harvested from *T. koksaghyz* root in 90 µL REB buffer [28]. Subsequently, total RNA was extracted using the innuPREP RNA Mini Kit (Analytik Jena, Jena, Germany) according to the manufacturer’s instructions. Full-length cDNA was synthesized from 500 ng total RNA using PrimeScript RT Master Mix (TaKaRa, Clontech, Saint-Germain-en-Laye, France) according to the manufacturer’s instructions. Quantitative real-time PCR analysis was performed as previously described [25]. Oligonucleotide sequences are shown in Table S5. Primer efficiencies and amplification factors are summarised in Table S6.

### 4.4. Triterpene Analysis

Whole *T. koksaghyz* roots were frozen in liquid nitrogen, freeze dried, and crushed to a fine powder. For triterpene analysis, 100 mg of dry root material was used for saponification by adding 20 mL of methanol containing 6% potassium hydroxide and heating to 80 °C for 2 h. As an internal standard, 100 µL of betuline (2.5 mg/mL stock solution in acetone) was added. Samples were extracted three times with one volume of hexane. The hexane phases were pooled, evaporated and the samples were dissolved in 1 mL of acetone. For the fast triterpene extraction, 1 mL of acetone was added to approximately 10 mg of dry root material and incubated over night at 45 °C. The acetone extract was reduced to 100 µL and 0.5 µL was used for GC–MS analysis. To extract triterpene from NR, 2 g of dry root powder were mixed with 20 mL of water in a 50 mL reaction tube and a mixture of steel beads were added. The sample was vortexed for 30 min, and the floating rubber were transferred into a new tube, washed in fresh water and dried over night at 40 °C. For triterpene extraction, 25 mg of the resulting NR was mixed with 2.5 mL acetone and 100 µL of internal betuline standard (2.5 mg/mL stock solution) and incubated by rotating over night at 40 °C. The resulting extract was dried and dissolved in 1 mL of acetone. Triterpene analysis was performed with a GC–MS–QP 210 Ultra system (Shimadzu, Duisburg, Germany) equipped with a Rxi^®^-5ms column (Restek GmbH, Bad Homburg, Germany). (Zero point five microlitres) Injection was performed with 0.5 µL of the extracts using split modus (1:10) at an injector and interface temperature of 260 °C. The GC temperature programme was as follows: 120 °C for 3 min, temperature gradient of 15 °C per minute up to 330 °C, 330 °C for 10 min. Electron ionisation (EI) in the MS was set to 70 eV. Peak integration and identification was performed with the LabSolution software (Shimadzu, Duisburg, Germany) using a NIST library (NIST = National Institute of Standards and Technology) or by analysing the corresponding standards obtained from Extrasynthese (Genay, France). Quantification was performed in relation to the internal standard. Retention indices for the triterpene compounds were determined using the LabSolution software in relation to a C8–C40 alkane calibration standard (Sigma-Aldrich, Taufkirchen, Germany) using the same operating conditions. For quantification in the case of the fast triterpene extraction, the relation of triterpene end-products and sterols was calculated.

### 4.5. Quantitative Determination of Poly(cis-1,4-isoprene) Levels by ^1^H -NMR Spectroscopy

For the quantification of the poly(*cis*-1,4-isoprene), the highly standardized and automated lifespin system (lifespin GmbH, Regensburg, Germany) was used. Two hundred milligrams of powdered and dried roots were extracted with 1500 µL of a toluene mixture (containing 10% Toluene-d8, TMS and 16 mM 2,6-dimethoxyphenol as an internal standard) for 16 h at 20 °C with continuous shaking at 1000 rpm. Cell debris were removed by centrifugation (14,500 rpm, 10 min). Six hundred microlitres of the supernatant were transferred to 5-mm NMR tubes and analysed by ^1^H-NMR spectroscopy using a Bruker AVANCE III 400 MHz spectrometer equipped with a 5-mm BBI probe-head. All measurements were obtained in full automation mode using lifespin software and measurement protocols. The temperature was equilibrated to 298 K. The ^1^H-NMR data were acquired using a one-dimensional ^1^H-NMR pulse programme with a 90° pulse and a relaxation delay of 20 s. Processing of the raw data was performed by lifespin software, including correction of the phase and the base-line. For quantification, the software integrated the signals of the C5 methyl signal for poly(*cis*-1,4-isoprene) at 1.75 ppm and the methyl signal of DMOP at 3.34 ppm. For quality control, in each run the integrals of DMOP were checked against calibrator samples and a control sample with a known poly(*cis*-1,4-isoprene) concentration.

## 5. Patents

A submitted patent application relates to this study: “Method to obtain low triterpene/triterpenoid containing natural rubber latex” (FHG16527EP).

## Figures and Tables

**Table 1:** Isoprenoid analysis of acetone extracts from NR obtained from *TkOSC1/TkOSC*-RNAi and WT root material by GC–MS. Extracts were analysed from the material of two plants of sL2, one plant of sL4, one plant of gL1 and two plants of gL2, respectively, compared to WT material (*n* = 3); asterisks denote statistical significance compared to control (two-tailed t test, * *p* < 0.05, ** *p* < 0.01); precursor: triterpene (squalene and 2,3-oxidosqualene) and sterol precursor (cycloartenol and 24-methylene cycloartanol); sterols: campesterol, stigmasterol and sitosterol; pentacyclic triterpenes: taraxasterol, α- and β-amyrin, lup-19(21)-en-3-ol, lupeol, taraxerol and four unknown triterpenes (free triterpenes (alcohols) as well as acetate derivatives); total triterpenes: precursor, sterols and pentacyclic triterpenes; single triterpene levels are shown in Table S3.

mg g^−1^ NR	WT	TkOSC1-RNAi sL2/sL4	TkOSC-RNAigL1/gL2
precursor	1.0 (±0.7)	3.5 (±0.9)*	8.6 (±1.2) **
sterols	1.2 (±0.2)	2.1 (±0.6)	1.4 (±0.1)
pentacyclic triterpenes	56.6 (±0.6)	33.4 (±4.0)*	16.0 (±2.9) **
total triterpenes	58.9 (±1.0)	39.1 (±5.4)*	26.1 (±1.6) **
pentacyclic triterpene reduction		−41.0%	−71.6%
total triterpene reduction		−33.6%	−55.6%

**Table 2:** Isoprenoid analysis of acetone extracts from NR isolated from *TkOSC*-RNAi and NIL roots of the T1 generation. Extracts in duplicate or triplicate were made from the material of three NIL-, two gL2-, and three gL3-plants, respectively; asterisks denote statistical significance compared to control (two-tailed t test, ** *p* < 0.01); precursor: triterpene (squalene and 2,3-oxidosqualene) and sterol precursor (cycloartenol and 24-methylene cycloartanol); sterols: campesterol, stigmasterol and sitosterol; pentacyclic triterpenes: taraxasterol, α- and ß-amyrin, lup-19(21)-en-3-ol, lupeol, taraxerol and four unknown triterpenes (free triterpenes (alcohols) as well as acetate derivatives); total triterpenes: precursor, sterols and pentacyclic triterpenes; single triterpene levels are shown in Table S4.

mg g^−1^ NR	WT	gL2	gL3
precursor	1.0 (±0.2)	6.8 (±0.6) **	3.3 (±1.2) **
sterols	0.9 (±0.2)	0.9 (±0.1)	0.9 (±0.1)
pentacyclic triterpenes	57.3 (±5.9)	11.8 (±1.4)**	15.4 (±2.5) **
total triterpenes	59.3 (±6.0)	19.5 (±1.7) **	19.6 (±1.5) **
pentacyclic triterpene reduction		−79.5%	−73.1%
total triterpene reduction		−67.0%	−66.9%

## Supplementary Materials

The following are available online, Table S1: Triterpene content of root material from *TkOSC1-*RNAi plants compared to WT material, Table S2: Triterpene content of root material from *TkOSC-*RNAi plants compared to WT material, Table S3: Triterpene content in NR acetone extracts from *TkOSC1/TkOSC-*RNAi plants compared to WT material, Table S4: Triterpene content in NR acetone extracts from *TkOSC*-RNAi plants in T1-generation compared to NIL material, Table S5: Sequences of oligonucleotides used for cloning and quantitative RT-PCR, Table S6: Primer efficiency and amplification factors for cDNA obtained from *T. koksaghyz* mRNA.
