# Supplementary material for: Oxidosqualene Cyclase Knock-Down in Latex of Taraxacum koksaghyz Reduces Triterpenes in Roots and Separated Natural Rubber
Source: Molecules. 2019 Jul 25;24(15):2703. doi: 10.3390/molecules24152703 (PMC6696514; doi:10.3390/molecules24152703)
Supplement: Supplementary file 1 [file molecules-24-02703-s001.pdf]

Table S1. Triterpene content of root material from TkOSC1-RNAi plants compared to WT material. Triterpene compounds were identified and quantified by GC-MS using the LabSolution software; quantification was performed in relation to the internal standard; corresponding retention indices (RI) were determined in relation to a C8–C40 alkane calibration standard; mean values of four (WT) or five (L2) plants (including three technical replicates for each plant) and the corresponding standard deviation; L4, n=2 (including three technical replicates); asterisks denote statistical significance compared to control (two-tailed t test, * = $p < 0.05$, ** = $p < 0.01$)

	RI	WT	sL2	sL4
**precursors**				
squalene	2843	0.01 (±0.00)	0.01 (±0.01)	0.01
2,3-oxidosqualene	2965	0.04 (±0.01)	0.07 (±0.05)	0.05
cycloartenol	3478	n.d.	0.11 (±0.02)	0.05
24-methylene cycloartanol	3536	n.d.	0.05 (±0.00)	0.05
**sterols**				
campesterol	3313	0.07 (±0.01)	0.07 (±0.01)	0.07
stigmasterol	3343	0.22 (±0.05)	0.21 (±0.02)	0.20
sitosterol	3405	0.21 (±0.01)	0.24 (±0.04)	0.25
**pentacyclic triterpenes**				
taraxerol and unknown triterpene	3448	0.70 (±0.10)	0.83 (±0.19)	0.76
ß-amyrin	3465	0.73 (±0.06)	0.28 (±0.07)**	0.17
lupeol	3510	0.12 (±0.03)	0.20 (±0.08)	0.14
α-amyrin and lup-19(21)-en-3-ol	3516	0.70 (±0.06)	0.58 (±0.13)	0.38
unknown triterpene	3550	0.06 (±0.01)	0.06 (±0.01)	0.04
unknown triterpene	3590	0.48 (±0.06)	0.50 (±0.09)	0.34
unknown triterpene	3603	0.43 (±0.04)	0.21 (±0.06)**	0.13
taraxasterol	3615	1.36 (±0.14)	0.37 (±0.14)**	0.23

Table S2. Triterpene content of root material from TkOSC-RNAi plants compared to WT. Triterpene compounds were identified and quantified by GC-MS using the LabSolution software; quantification was performed in relation to the internal standard; corresponding retention indices (RI) were determined in relation to a C8–C40 alkane calibration standard; mean values of three plants for all transgenic lines and WT (including three technical replicates for each plant) and the corresponding standard deviation; asterisks denote statistical significance compared to control (two-tailed t test, * = p < 0.05, ** = p < 0.01).

	RI	WT	gL1	gL2	gL3
**precursors**					
squalene	2843	0.01 (±0.00)	0.03 (±0.02)	0.03 (±0.01)	0.02 (±0.00)
2,3-oxidosqualene	2965	0.03 (±0.01)	0.17 (±0.05)*	0.18 (±0.12)	0.05 (±0.02)
cycloartenol	3478	n.d.	0.18 (±0.03)	0.17 (±0.04)	0.11 (±0.00)
24-methylene cycloartanol	3536	n.d.	0.16 (±0.04)	0.14 (±0.03)	0.12 (±0.02)
**sterol**s					
campesterol	3313	0.07 (±0.01)	0.07 (±0.00)	0.07 (±0.01)	0.06 (±0.01)
stigmasterol	3343	0.22 (±0.06)	0.23 (±0.00)	0.26 (±0.01)	0.22 (±0.01)
sitosterol	3405	0.22 (±0.01)	0.25 (±0.02)	0.23 (±0.01)	0.22 (±0.03)
**pentacyclic triterpenes**					
taraxerol and unknown triterpene	3448	0.69 (±0.12)	0.86 (±0.07)	0.84 (±0.15)	0.71 (±0.03)
ß-amyrin	3465	0.71 (±0.06)	0.27 (±0.03)**	0.25 (±0.07)**	0.19 (±0.00)**
lupeol	3510	0.12 (±0.03)	0.13 (±0.02)	0.10 (±0.02)	0.10 (±0.03)
α-amyrin and lup-19(21)-en-3-ol	3516	0.69 (±0.08)	0.67 (±0.12)	0.67 (±0.27)	0.43 (±0.01)**
unknown triterpene	3550	0.06 (±0.01)	0.06 (±0.02)	0.07 (±0.02)	0.06 (±0.01)
unknown triterpene	3590	0.47 (±0.07)	0.56 (±0.14)	0.63 (±0.18)	0.51 (±0.10)
unknown triterpene	3603	0.42 (±0.04)	0.15 (±0.01)**	0.12 (±0.03)**	0.11 (±0.01)**
taraxasterol	3615	1.31 (±0.11)	0.18 (±0.02)**	0.10 (±0.02)**	0.15 (±0.03)**

Table S3. Triterpene content in NR acetone extracts from TkOSC1/TkOSC-RNAi plants compared to WT. Triterpene compounds were identified and quantified by GC-MS using the LabSolution software; quantification was performed in relation to the internal standard; corresponding retention indices (RI) were determined in relation to a C8–C40 alkane calibration standard; mean values of three plants for both RNAi-constructs and WT, and the corresponding standard deviation; asterisks denote statistical significance compared to control (two-tailed t test, * = $p < 0.05$, ** = $p < 0.01$).

mg g^{-1} NR	RI	WT	TkOSC1-RNAi sL2/sL4	TkOSC-RNAi gL1/gL2
**precursors**				
squalene	2843	0.59 (±0.53)	0.54 (±0.39)	0.58 (±0.23)
2,3-oxidosqualene	2965	0.43 (±0.17)	1.16 (±0.53)	4.55 (±0.95)*
cycloartenol	3478	n.d.	1.48 (±0.46)	2.06 (±0.11)
24-methylene cycloartanol	3536	n.d.	0.37 (±0.30)	1.45 (±0.06)
**sterol**s				
campesterol	3313	0.15 (±0.03)	0.23 (±0.07)	0.16 (±0.02)
stigmasterol	3343	0.43 (±0.08)	0.59 (±0.19)	0.51 (±0.05)
Sitosterol	3405	0.67 (±0.04)	1.32 (±0.38)	0.78 (±0.02)
**pentacyclic triterpenes**				
taraxerol and unknown triterpene	3448	1.84 (±0.91)	3.65 (±0.85)	1.93 (±0.21)
ß-amyrin	3465	6.13 (±0.60)	3.69 (±0.95)*	1.83 (±0.32)**
Lupeol	3510	1.85 (±0.91)	3.53 (±0.29)	1.30 (±0.24)
α-amyrin and lup-19(21)-en-3-ol	3516	4.74 (±0.34)	7.89 (±2.02)	6.46 (±1.40)
ß-amyrin acetate	3562	3.17 (±0.47)	0.47 (±0.27)**	n.d.
unknown triterpene	3550	0.08 (±0.01)	0.28 (±0.12)	0.17 (±0.02)
unknown triterpene	3590	0.62 (±0.06)	1.49(±0.43)	1.43 (±0.24)
unknown triterpene	3603	8.27 (±0.07)	5.54 (±0.97)*	1.09 (±0.26)**
taraxasterol	3615	22.58 (±1.86)	6.97 (±0.77)**	1.85 (±0.59)**
unknown triterpene acetate	3695	2.41 (±0.40)	0.54 (±0.20)**	n.d.
taraxasterol acetate	3707	4.90 (±1.14)	0.33 (±0.24)**	n.d.

Table S4. Triterpene content in NR acetone extracts from TkOSC-RNAi plants in T1-generation compared to NIL. Triterpene compounds were identified and quantified by GC-MS using the LabSolution software; quantification was performed in relation to the internal standard; corresponding retention indices (RI) were determined in relation to a C8–C40 alkane calibration standard; mean values of 2-3 extracts from of three NIL-, two gL2-, and three gL3-plants, respectively, and the corresponding standard deviation; asterisks denote statistical significance compared to control (two-tailed t test, ** = $p < 0.01$).

mg g^{-1} NR	RI	WT	gL2	gL3
**precursors**				
squalene	2843	0.34 (±0.11)	0.50 (±0.04)	0.40 (±0.05)
2,3-oxidosqualene	2965	0.69 (±0.09)	5.47 (±0.52)**	2.47 (±1.10)**
cycloartenol	3478	n.d.	0.52 (±0.08)	0.33 (±0.06)
24-methylene cycloartanol	3536	n.d.	0.34 (±0.06)	0.15 (±0.06)
**Sterol**s				
campesterol	3313	0.08 (±0.01)	0.10 (±0.01)	0.08 (±0.01)
stigmasterol	3343	0.27 (±0.06)	0.42 (±0.04)	0.30 (±0.03)
sitosterol	3405	0.59 (±0.08)	0.42 (±0.05)	0.47 (±0.10)
**pentacyclic triterpenes**				
taraxerol and unknown triterpene	3448	2.75(±0.37)	1.72 (±0.17)	1.80 (±0.31)
ß-amyrin	3465	8.81 (±0.68)	1.84 (±0.21)**	2.67 (±0.47)**
lupeol	3510	1.00 (±0.18)	0.54 (±0.09)**	0.61 (±0.19)**
α-amyrin and lup-19(21)-en-3-ol	3516	6.38 (±0.53)	3.88 (±0.72)	3.95 (±0.65)
ß-amyrin acetate	3562	5.21 (±2.56)	n.d.	n.d.
unknown triterpene	3550	0.10 (±0.02)	0.19 (±0.02)	0.15 (±0.01)
unknown triterpene	3590	0.38 (±0.06)	0.77 (±0.10)	0.64 (±0.05)
unknown triterpene	3603	7.99 (±1.04)	1.03 (±0.12)**	1.67 (±0.37)**
taraxasterol	3615	16.85 (±2.58)	1.80 (±0.08)**	3.95 (±0.55)**
unknown triterpene acetate	3695	2.45 (±1.25)	n.d.	n.d.
taraxasterol acetate	3707	5.36 (±2.70)	n.d.	n.d.

Table S5. Sequences of oligonucleotides used for cloning and quantitative RT-PCR.

oligo	sequence (5'→3')
TkOSC1-RNAi-fwd-NcoI	AAACCATGGGCGGAATTGATCTTATAAGCG
TkOSC1-RNAi-rev-XhoI	AAACTCGAGATCCCAAGCTTGAATCGCAC
TkOSC-RNAi-fwd-NcoI	AAACCATGGAACAAGAAAATGGTTCTTGG
TkOSC-RNAi-rev-XhoI	AAACTCGAGTCTCCAAGCGCCCATAGCGG
TkEF1alpha-fw-realtime	CGAGAGATTCGAGAAGGAAGC
TkEF1alpha-rv-realtime	CTGTGCAGTAGTACTTGGTGG
TkLUP-fw-realtime	GCTGACCACCACCAACAACCAC
TkLUP-rv-realtime	AGCACGTTCCTCTTCGGTTCCAG
TkOSC1-fw-realtime	ACTCCTCCCTTGATAATTGCCC
TkOSC1-rv-realtime	TTGTGCTTCTGCCTGATATATAGAAC
TkOSC2-fw-realtime	CCGGTGAGAAGGTGGAAGTT
TkOSC2-rv-realtime	GGAACCGGTACCTCCCAAAC
TkOSC3-fw-realtime	TCCATCCAAACCACAGAAAAG
TkOSC3-rv-realtime	ATGAAGCATACTCCCCAATAAC
TkOSC4-fw-realtime	CCGACAATTCGTAAGGCCACTG
TkOSC4-rv-realtime	TGTTTGCACCACGTTCGACC
TkOSC5-fw-realtime	GAAACACAACTAGAAGATGGCGGT
TkOSC5-rv-realtime	CATAGCCCATGAAGTGTGCACT
TkOSC6-fw-realtime	GGTCATAGCACCATGTTTGGG
TkOSC6-rv-realtime	GGTGACTGAGCCATGATCCAGG
TkRP-fw-realtime	CGTCGATCTCAAGGATGTTGTC
TkRP-rv-realtime	GGAGCTTTGAGAAGAACCAACG

Table S6. Primer efficiency and amplification factors for cDNA obtained from *T. koksaghyz* mRNA. The values were calculated using the Bio-Rad CFX Manager v3.1 software (Bio-Rad Laboratories Inc., Hercules, CA, USA) and the qPCR primer efficiency calculator provided by Thermo Fisher Scientific (http://www.thermoscientificbio.com/webtools/qpcrefficiency/).

oligo pair	efficiency	amplification factor (66°C)
TkLUP-realtime	99.17%	1.99
TkOSC1-realtime	100.16%	2.00
TkOSC2-realtime	101.87%	2.02
TkOSC3-realtime	109.67%	2.10
TkOSC4-realtime	108.54%	2.09
TkOSC5-realtime	106.95%	2.07
TkOSC6-realtime	94.39%	1.94
TkEF1alpha-realtime	104.48%	2.04
TkRP-realtime	105.44%	2.05
